# Camel milk protectiveness toward multiple liver disorders: A review

**DOI:** 10.3389/fnut.2022.944842

**Published:** 2022-09-15

**Authors:** Khunsha Shakeel, Roshina Rabail, Iahtisham-Ul-Haq, Sabrina Sehar, Asad Nawaz, Muhammad Faisal Manzoor, Noman Walayat, Claudia Terezia Socol, Cristina Maria Maerescu, Rana Muhammad Aadil

**Affiliations:** ^1^National Institute of Food Science and Technology, University of Agriculture, Faisalabad, Pakistan; ^2^Kauser Abdulla Malik School of Life Sciences, Forman Christian College (A Chartered University), Lahore, Pakistan; ^3^Shenzhen Key Laboratory of Marine Microbiome Engineering, Institute for Advanced Study, Shenzhen University, Shenzhen, China; ^4^School of Food and Biological Engineering, Jiangsu University, Zhenjiang, China; ^5^College of Food Science and Technology, Zhejiang University of Technology, Hangzhou, China; ^6^Department of Genetics, University of Oradea, Oradea, Romania

**Keywords:** camel milk, liver disorders, hepatitis, non-alcoholic fatty liver disease, cirrhosis, hepatocellular carcinoma

## Abstract

Camel milk is known as the white gold of the desert because it contains within it a variety of nutrients which play a key role in the human diet. The health benefits of camel milk have been described for a variety of diseases such as diabetes, kidney disease, hepatitis, etc. including improved overall survival. A major health burden worldwide is liver diseases, and the ninth leading cause of death in Western countries is due to liver cirrhosis. Treatment is mostly ineffective for cirrhosis, fatty liver, and chronic hepatitis which are the most common diseases of the liver; furthermore current treatments carry the risk of side effects, and are often extremely expensive, particularly in the developing world. A systematic review of studies was performed to determine the association of consumption of camel milk on multiple diseases of the liver. The impact of camel milk on the laboratory tests related to the liver disorders, viral hepatitis, non-alcoholic fatty liver disease (NAFLD), cirrhosis, and hepatocellular carcinoma (HCC) were evaluated. The consumption of camel milk was accompanied by modulation of the values of serum gamma-glutamyl transferase, aspartate aminotransferase, and alanine aminotransferase in persons who are at risk of liver disease. In the patients with chronic liver disease, it was observed that they have low rates of mortality and low chances of progression to cirrhosis when they consume camel milk. Therefore, in patients with liver diseases, the addition of camel milk to their normal daily diet plan should be encouraged. In this review, camel milk's impact on the different kinds of liver diseases or any disorder associated with liver functioning was evaluated. Camel milk has a therapeutic as well as a preventive role in the maintenance and improving the metabolic regulations of the body.

## Introduction

Milk is considered one of the most important foods for humans and animals, and acts as a complete diet due to its crucial components such as carbohydrates, proteins, fats, vitamins, and minerals (1–3). Milk composition is highly dependent, which can be greatly affected by many factors such as animal's health status, especially the mammary gland health, photoperiod effect of different seasons, animal's diet (for example a higher concentrate intake during dry season), genetic factors, and the temperature of milk storage (4–6). Among varieties of milk available, camel milk is also known as the “white gold” of the desert as it contains essential nutrients that play an important role. In desert areas worldwide, Dromedary camels (*Camelus dromedarius*) are habitant, and the milk is available in the form of many products, of which cheese, powdered camel milk, coffee, and ice cream are sold in many developed countries (7, 8). Its milk is also available commercially at low prices. The production of camel milk has increased 4.6 times globally and the production level has reached 2.9 million tons between 1961 and 2017 (9). Camel milk has a great nutritive value, as well as the value of its functional ingredients in it (10). Table 1 highlights the nutritional components of camel milk. Like other kinds of milk, it also consists of all essential nutrients. The composition of cow milk is compatible with that of camel milk. It contains more zinc, iron, vitamin C, and E as compared to other kinds of milk (11). It has an essential role in the improvement of the immune system. It can fight against major diseases and this attribute is because it contains major proteins such as lactoferrin, peptidoglycan, antibodies immunoglobulins and some enzymes such as lysozymes and lactoperoxidase are present. It may improve our mechanism of defense by improving the immune system if it is consumed on daily basis. The amount of sugar and cholesterol is very low, which is why it is considered superior to all other ruminant milk. It has a miraculous impact on the health of human beings as it contains insulin, and vitamin C is present in a very high amount (12). The vitamins contained play an important role as it has antioxidant activity as well as a role in the regulation of damage caused by destructive substances (13). This problem was handled with scientific research in which the antioxidant activity was determined to prove the remedial properties of camel milk (14).

**Table 1:** The nutritional components of camel milk.

**Nutrients in camel milk**	**Values**	**References**
Lactose	49.8 g/l	(15)
Fat	35.6 g/l	
Polyphenols	35.45 mg GAE/1	
Protein	32.6 g/l	
Flavonoids	29.05 mg EQ/1	
Vitamin C	27.53 mg/l	
Ashes	8.06 g/l	

The whey proteins, casein proteins, and lactic acid bacteria from the camel milk were evaluated to determine its antioxidant activity; regulation of the immune system, role in inflammation, activities related to probiotic properties, and anti-microbial were studied (16–19). Table 2 elaborated the mechanism of action behind such bioactive components of camel milk in the body against various biomarkers. Because of its antioxidant capability, camel milk is used to remove the side effects caused by chemo- and radiotherapy, as well as being used in the treatment of many types of cancers (20). Studies have shown that camel milk produces important beneficial effects on human health, as it contains low levels of β-lactoglobulin, making it suitable for consumption by persons who cannot tolerate lactose in their meals (21).

**Table 2:** Mechanism of action of bioactive components of camel milk.

**Active component**	**Mechanism**	**References**
Lactoferrin	• Monocytes or macrophages and granulocytes are upregulated • Vital function during the early phases of viral infection • Takes over the antiviral response • Improves the level of alkaline phosphatase (ALP) and aspartate aminotransferase (AST) biomarkers	(22, 23)
Whey protein concentrate	• Minimizes the effect of viral load reducing • Serum albumin normalized • Improvement in the neutrophils' phagocytic function • Serum levels of alanine aminotransferase (ALT), ICAM-1, IL-2 reduced	(24)
Immunoglobulin	• Ability to enter ins tissues and cells • The enzyme activity of bacteria or viruses is normalized	(25)
Ascorbic acid	• Helps in improving liver function	(26)
Vitamin B, C, and E	• Act as an antioxidant • Plays a role in the reduction of hepatic fat accumulation • Oxidative stress of systemic and hepatic systems is reduced	(27)
Low lipid content	• The high value of L-carnitine • Absorption of cholesterol decreases	(28)
Camel milk proteins	• Protection of non-alcoholic fatty liver diseases • Reduces inflammatory angiogenesis	(29, 30)
Lactoferrin	• Inhibitory effect on HCV • In chronic hepatitis C patients, lactoferrin improves HCV RNA and ALT levels	(31)
Magnesium	• Delays the aging process of skin • Major role in the hair growth	(32)
Zinc	• Alcoholic liver disease is improved *via* processes like • Programmed death of hepatocytes • Reduction of endotoxemia • Proinflammatory cytokines decreased	(33)

The popularity of camel milk is due to its source nutrient content. In some areas of Africa and Asia, it is consumed as a major part of their staple diet and it is assumed that it may have a role in the promotion of good health. For this reason, the utilization of camel milk in the form of fresh milk or sour milk is recommended so that the complications of liver or kidney disease such as increased oxidative stress, delayed wound healing, and high levels of cholesterol in the blood can be controlled. Therefore, an effort is made here to compile the latest available literature from the last 10 years studies were done on the health promotion and protectiveness of camel milk toward liver disorders of various kinds.

## Liver disorders

In the functioning of the human body, the liver plays a vital role. It is responsible for detoxification and it metabolizes various components of food that enters our body (34). It is involved in the metabolic, vascular, secretory, immunological, and excretory functions of the human body. The metabolism of key nutrients such as carbohydrates, proteins, and fats is also served by the liver (35). The control of the flow of substances that are absorbed from the digestive system and then distributed to blood circulation to play its key role in the targeted organ site is the primary function of the liver. The total loss in its functions can cause sudden death which shows its great importance in the human body (36). So, it is very important to keep the liver healthy, otherwise, it may cause several fatal diseases of the liver including liver cirrhosis, hepatitis, fatty liver disease, alcoholic liver disease, etc. are included (37).

Globally, hepatic diseases are a major human health problem, in which the rates of morbidity and mortality are high. Many things play role in the cause of injury to the liver such as alcohol, drugs, viruses, and lipopolysaccharides type of bacteria (38). There are different mechanisms as well as many risk factors present that have a role in the expansion of healthy liver into liver fibrosis, cirrhosis, failure of the liver, and the many related complications, which in some cases progress to liver cancer. There are many toxins taken into the liver, such as high intake of alcohol, heavy metals, and organic and inorganic solvents, and when exposed these can lead to the production of many free radicals in the liver that may develop into hepatic lesions, which include liver cirrhosis, hepatitis, and liver carcinoma (39). The exposure of the liver to radiation may be accidental or therapeutic, but the damage will lead to the improper functioning of the liver which may include the excretion of harmful waste products, production of bile, storage of glucose in the form of glycogen, and synthesis of protein (40). There are multiple diseases or problems of the liver, for example, hepatitis, alcoholic liver disease, hepatotoxicity, liver carcinoma, cirrhosis, etc. Therefore, the effect of camel milk on these hepatic diseases has been discussed one by one to highlight the liver protective potential.

## Hepatoprotective effect of camel milk

Table 3 explains the scientific studies reporting the hepatoprotectiveness of camel milk. One such study was done to determine the impact of camel milk on the enzymes of the liver, total proteins, and histology of poloxamer 407 induced hyperlipidemic rats when it is orally given to the Wister rats having a hyperlipidemic problem. Thirty male Wister rats whose weight was between 150 and 200 g were divided into six groups having five rats each. To evaluate the levels of alkaline phosphatase (ALP), alanine aminotransferase (ALT), aspartate aminotransferase (AST), total protein, albumin value, globulin, the ratio of albumin and globulin, samples of rats' blood and liver tissues were taken after 3 weeks. All the groups which were treated with camel milk showed a substantial (*p* < 0.05) decrease in the levels of ALT and AST. There is a markable reduction in the levels of total protein content, and globulin in the groups which are given with the camel milk at 250 and 1,000 mg/kg, and there is an increase in the Albumin/Globulin ratio in all the groups that are treated with camel milk (41).

**Table 3:** Scientific studies explain the hepatoprotectiveness of camel milk.

**Year**	**Camel milk dosage**	**Subject**	**Type of liver disease**	**Materials and methods**	**Results**	**References**
2022	Camel milk and camel urine	24 Mice divided into 4 groups	Hepatotoxicity	G1 = control, G2 = positive CCL_4_, G3 = camel milk (100 ml/day/cage) injected with CCL_4_, G4 = camel Urine (100 ml/day/cage) injected with CCL_4_	There is a defensive function of camel milk and camel urine against hepatotoxicity induced with CCL_4_	(42)
2021	Camel's milk	Adult female Sprague Dawley rats = 100	Hepatotoxicity	G1 = Oral dose of MXC 200 mg/kg BW (methoxychlor-induced liver damage), G2= (100 mL/day) camel milk given for 6 or 12 months, G3: daily dose of (100 mL/day) for 6 or 12 months	There is protecting role of camel milk against methoxychlor-induced liver damage	(43)
2021	Probiotics from camel milk	Mice = 40	Liver injury	Model groups = skimmed camel milk, Metformin group= 0.3 g per kg BW metformin. Probiotic groups= probiotics from camel milk are given in a low and high dose	The liver and kidney damage is improved with camel milk probiotics that regulate lipid metabolism, and protection in mice	(44)
2021	Camel whey protein hydrolysates (CWPH)	TAA- toxicity induced male Wistar albino rats=35	Hepatorenal failure	G1 = 5 mL sterile distilled water; G2 = TAA (200 mg/kg BW), G3 = TAA (200 mg/kg BW) + CWPH (50 mg/kg BW/day orally, G4 = TAA (200 mg/kg BW) + CWPH (100 mg/kg BW/day orally, G5 = TAA (200 mg/kg BW) + CWPH 200 mg/kg BW/day orally	CWPH has hepatorenal protective properties	(45)
2021	Camel milk antibodies	Male Wister rats	Hepatocellular carcinoma	Hepatocarcinoma induced by DENA + CCl_4_ Then camel milk antibodies CM-IgG (100 mg/kg, orally) given	IgG from camel milk in the removal of dysfunction of liver cells oxidative stress induced by DENA	(46)
2021	Camel milk	Albino rats =96	Hepatotoxicity	G1: saline solution, All remaining groups: camel milk 2, 4, and 6 ml/100 g BW, respectively	Camel milk ingestion resulted in restorations of functions of kidney and liver biomarkers	(11)
2020	Camel milk	Mice = 24	Alcoholic liver disease	G1 (control group) = normal diet + 0.3 mL water, G2 (ethanol group) =normal diet + 0.3 mL water, G3 (Camel milk treatment group) = ethanol + camel milk and skimmed camel milk powder	Camel milk protects against liver injury caused by alcohol	(47)
2019	Camel milk lactoferrin	Male Sprague Dawley rats = 75	Hepatic fibrosis	CCL_4_+ 40% CCL_4_ (mix with olive oil) at 200 uL/100 g BW. Among all groups 30, 60, and 90 mg/kg/BW given with standard diet + lactoferrin orally Control group = standard diet throughout the study	Camel milk lactoferrin improved blood levels of ALP, AST, bilirubin, serum urea, and serum creatinine levels	(48)
2019	LAB from camel milk	Mice	Liver disease	Mice were given six strains of LAB for 7 weeks	Probiotics from camel act as a liver injury inhibitor	(38)
2018	Camel milk + NSO	Female albino Wister rats=30	Hepatotoxicity	G1 = normal control, G2 = toxic control, G3, G4, and G5 = camel milk, NSO, and NSO+ camel milk, respectively. Group VI = Unani medicine Jigreen	Protective effects of camel milk, and camel milk + *Nigella sativa* oil on the toxicity of liver and kidney in rats	(49)
2018	FCM	Male rats = 42	Non-alcoholic fatty liver disease	G1 = standard diet, G2 = HFDHFr to induce fatty liver disease The remaining five groups = HFDHFr + camel milk, (FCM) having non-encapsulated probiotic bacteria, FCM having microencapsulated probiotic without prebiotic, FCM containing microencapsulated probiotic and 1% ginger extract or FCM having microencapsulated probiotic and 10% beetroot extract, respectively	FCM containing microencapsulated probiotics with plant extract reduced the severity of fatty liver	(50)
2018	FCM	Female Wister mice = 56	Liver damage	Control mice= water+ standard diet G2: CCL_4_ in 0.3% olive oil, G3: FCM, G4: *R. officinalis*, G5 = *R. officinalis* + FCM, G6: firstly given with FCM then toxicated with CCL_4_, G7: Initially treated with *R. officinalis* then toxicated with CCL_4_, G8: Initially treated with FCM+ rosemary then toxicated with CCL_4_	FCM in combination + with *R. officinalis* extract is beneficial in reducing liver injury	(51)
2018	FCM	Human (adults)	Liver enzymes status	Overweight/obese adolescents were given camel milk 250 cc per day for 8 weeks, then diluted Cow milk yogurt 250 cc per day for 8 weeks or vice versa	FCM can be given as a functional food supplement	(52)
2018	Camel milk	Rats = 30	Hepatotoxicity	G1 = 0.5 ml normal saline, G2 = 3 g/kg/day ethanol, G3 = 1 mL/kg/day/orally camel milk, G4 = ethanol (3 g/kg/day) + camel milk (1 mL/kg/day), G5 = ethanol (3 g/kg/day) group	Camel milk has a protective and prophylactic effect against liver toxicity induced by ethanol	(53)
2017	Camel milk yogurt enriched with fig and honey	Male albino rats = 47	Steatohepatitis	G1 = +ve control MCDD, G2 = MCDD + Camel milk yogurt 30%, G3, G4, and G5 were given MCDD with 30% camel milk yogurt+ fig and honey, respectively	Protective effect on steatohepatitis	(54)
2017	Camel milk+ EVOO	Mice	Liver toxicity	G1 = Acetaminophen (500 mg/kg), G2 = camel milk (33 ml/kg), G3 = extra virgin olive oil (1.7 ml/kg), G4 = acetaminophen (500 mg/kg), G5 = camel milk +acetaminophen	Olive oil and camel milk have hepatoprotective action	(55)
2017	Camel milk given with drug cisplatin	Male rats = 56	Hepatocarcinogenesis	G1 = control group, G5, G6, G7, and G8 = DENA (200 mg/kg BW) and phenobarbitone (500 ppm) in drinking water, G2, G3, G4, G7, and G8 = Camel milk (5 mL/day) and cisplatin (5 mg/kg/3 weeks)	Reduction in the hepatocarcinogenesis with camel milk intake	(56)
2017	Camel milk + Peg IFN/RBV)	Human (adult patients) = 45	Chronic hepatitis C	G1 = (*n* = 23) Peg IFN/RBV in standard-dose, while G2 = (*n* = 22) Camel milk+ Peg IFN/RBV	Camel milk + Peg IFN/RBV improve the viral response + harmful effects of chronic hepatitis C are reduced	(57)
2017	Camel milk	Human= 17 patients (12 male + 5 females	Hepatitis C	Control = Three healthy adults included in study Participants = routine daily meals + camel milk 250 ml for 4 months	Camel milk decreased the viral load in the patient's sera	(58)
2017	Camel milk	Male Wister rats = 30	Altered liver enzymes	G1 = distilled water, G2 = induced with P407, G3 = induced with P407 then given atorvastatin (20 mg/kg), G4,5,6 = induced with P407 then camel milk 250, 500, and 1,000 mg/kg	Hepatoprotective effect of camel milk	(41)
2017	Camel milk in anti-tuberculous drugs	Male albino rats = 24	Hepatotoxicity	Rats were given a standard diet+ anti-tuberculous drugs+ camel milk G1 = normal diet + freshwater, G2 = anti-tuberculous drugs, G3 = 1 ml/kg BW of camel milk, G4 = 1 ml/kg BW of camel milk + Anti-tuberculous drugs	The toxicity and damage to the liver caused by anti-tuberculous drugs will be decreased with camel milk	(59)
2016	Camel milk + bee honey	Male rats = 36	Liver cirrhosis	G1 (control) = basal diet + tap water, G2 = basal diet + water intoxicated with CCL4, G3 = basal diet + camel milk, G4 = basal diet + camel milk + bee honey	Protecting effect of camel milk against CCL_4_-induced liver damage.	(60)
2016	Camel milk	Male adult Rats = 24	Liver injury	G1 = corn oil, G2 = water + CCL_4_ in a dose of 1 ml/kg in 50% corn oil, G3 = camel milk + corn oil, G4 = camel's milk+ CCL_4_ in a dose of 1 ml/kg 50% in corn oil.	Camel milk protects the liver and kidney against CCL_4−_generated oxidative stress and injuries	(61)

FCM, fermented camel milk; HFDHFr, high-fat diet and high fructose in water; Peg IFN/RBV, pegylated interferon and ribavirin; EVOO, extra virgin olive oil; LAB, lactic acid bacteria; NSO, nigella sativa oil; MCC, methionine choline-deficient diet; DENA, diethyl-nitrosamine.

There is a key role of lactic acid bacteria could play in the pathogenesis of liver diseases. In this study, the isolation of 107 strains of lactic acid bacteria from the products of Mongolian camel milk was done which was then identified as species, and the screening was performed to determine their probiotic properties. To study the protective effects of these strains on the acute injury of the liver that is induced by lipopolysaccharide (LPS)/D-galactosamine (D-GalN), six strains of lactic acid bacteria, which have strong bearing and bounding capacity were isolated. For 7 weeks, these six strains of LAB were given to the rats. The amount of aspartate aminotransferase (AST) and alanine aminotransferase (ALT) secretion in serum and liver, as well as the evaluation of expression of tumor necrosis factor (TNF) and interleukin (IL)-6 in the liver and intestines. This suggests the strength and role of probiotic and pharmacological value of *L. paracasei* subsp. In inflammation-based liver disease, paracasei is the inhibitor of liver injury (30).

**Figure 1:**
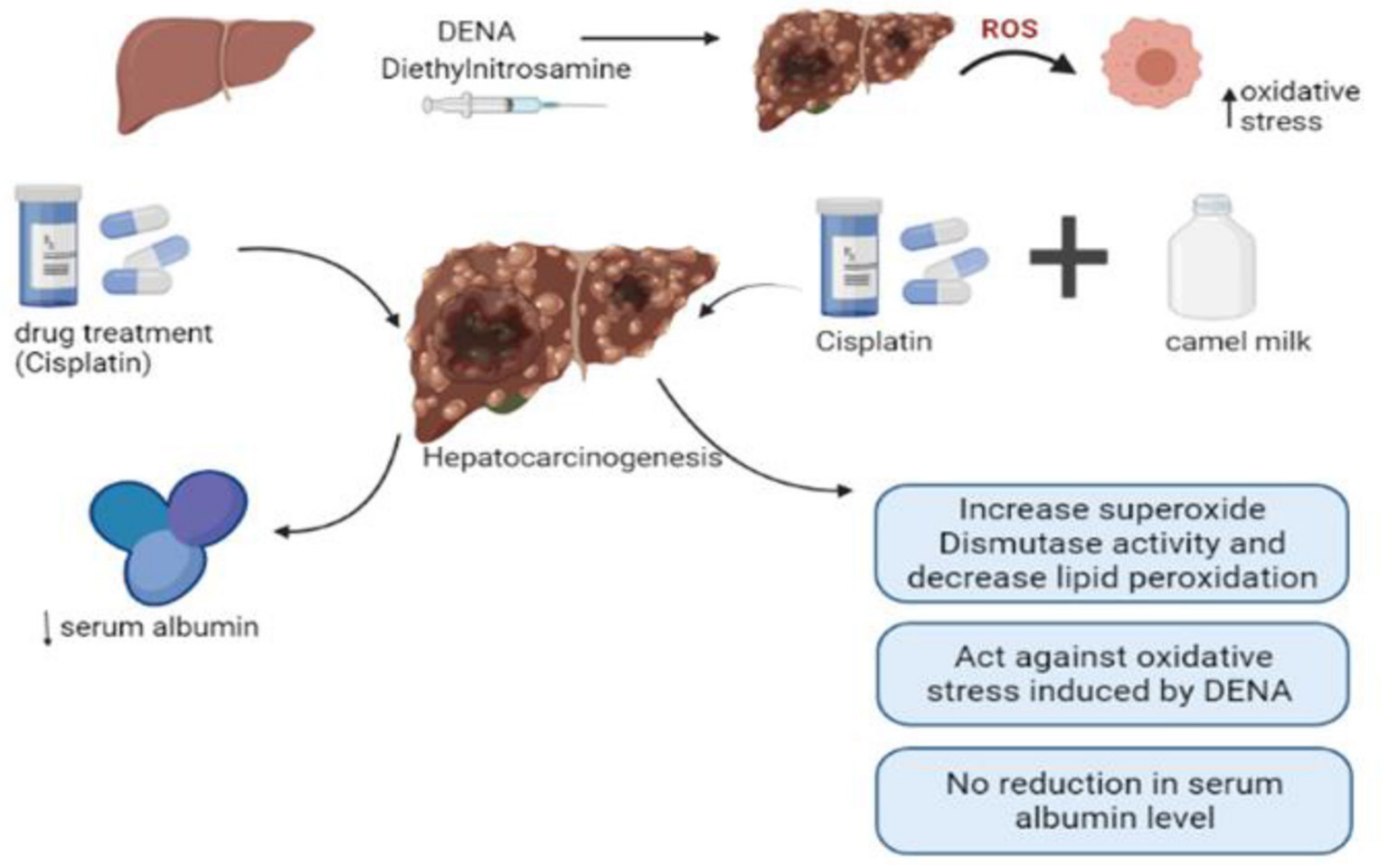
Effect of camel milk in combination with drug treatment.

In another study, rats injected with carbon tetrachloride (CCl4) showed upregulation of the expression of mRNA of hepatic IL-6 and renal IL-1β, TGF-β1, SREBP-1c, and caspase-6 and down-regulating the expressions of enzymes of anti-oxidation such as SOD, GST, and CAT in addition to hepatocellular vacuolation, mononuclear cell infiltration, and sinusoidal dilatation and renal glomerular atrophy, capsular space expansion, and adhesion between visceral and parietal layers of Bowman's capsule. Camel milk supplementation prior to and with CCL_4_ injection to rats attenuated CCL4-induced hepatic and renal inflammatory cytokines (IL-6, IL-1β, TGF- β1 SREBP-1c, and caspase-6), upregulated CCL4-suppressed anti-oxidative markers (SOD, GST, and CAT) and induced protective and regenerative mechanism (EPO and IL-10). Additionally, camel milk protects the liver and kidney from CCL4-induced histopathological changes in vacuolation. These results showed the mechanism of camel milk protection of the liver and kidney against CCL_4_-generated oxidative stress and injuries. The results of this study conclude the beneficial role of camel milk as a therapeutic adjuvant with drugs that are always associated with the production of oxidative stress that injured the liver and kidneys as anti-tumor drugs such as Cisplatin (62). Figure 1 explains the effect of camel milk in combination with drug treatment.

Likewise, a study was conducted to examine the effect of whey protein hydrolysates (WPH) of camel milk on the liver toxicity induced by thioacetamide (TAA) in rats. The assessment of enzymes of liver, protein and lipid profile, activities of antioxidant enzymes, function related to kidney, and pathological alterations in the liver was performed. There is a reduction in the antioxidant capacity, impaired functions related to liver and renal, and increased lipid content in blood in the animals having TAA toxicity. The CWPH conserved the hepatorenal functions, activities of antioxidative enzymes, and the lipid profile of the animals that are treated with CWPH, as well as countered the oxidative tissue damage caused by TAA. In addition, the CWPH can counteract the dysfunction of the hepatorenal. It showed that the camel whey protein has antihypertensive, antioxidant, and protective properties of hepatorenal after being hydrolyzed (37).

Moreover, a study was conducted in which the adolescents who are overweight or obese having complained of metabolic syndrome were given camel milk 250 cc per day for 8 weeks, then after 4 weeks of the gap, they were given diluted cow yogurt for the same time. After each period, the liver enzymes, anthropometric measurements, and serum lipids were assessed. Before each period, a food record of 3 days and a questionnaire related to physical activity were completed. Twenty-four participants of which 58% were girls, completed the study. In comparison to diluted cow yogurt, the levels of aspartate aminotransferase (AST), alanine aminotransferase (ALT), and the ratio of AST/ALT are decreased with the intake of fermented camel milk. The study infers that FCM may be used as a dietary supplement after observing its favorable effects in the related condition (44).

In another study, composite probiotics from camel milk (CPCM) were prepared after the separation of 4 lactobacilli and 1 saccharomycetes from traditional fermented cheese whey (TFCW). Then its effects on the metabolism of glucose and lipid, hepatorenal functions, and gut microbiota in mice were investigated. Free access to food and water was given to each mouse and disinfected-skimmed camel milk was provided to the model group, the metformin in the amount of 0.3 g per kg of BW was given to the metformin group. A low and high-dose CPCM was provided to the low and high probiotic groups. Histological examination of the pancreas and liver was completed with the help of optical microscopy on paraffin material. The tissue sections of the pancreas and liver were fixed with a 10% buffered solution of formalin and then histological preparations were made. In addition, it was observed that there is a regulation in the metabolism of the liver, improvement in the functions of fatty liver, renal, and gut microbiota functions with the help of composite probiotics in camel milk that acts by regulating the intestinal flora disturbance and protecting the functions of islets (36).

### Hepatitis

Hepatitis C virus (HCV) causes damage to the liver and is known as the major cause of it. Hepatitis is with medical therapies, though traditional and herbal medicines are also used. The prevalence of hepatitis globally is around 2.2%. In less developed countries like Pakistan, the rate of infection with hepatitis C grades on the second number ranging from in occurrence from 4.5 to 8% (63). It is the main cause of hepatic damage in developing countries such as Egypt as well, and globally it is considered a major health issue. Worldwide, around 130–180 million people are infected with HCV. It has been suggested that in the upcoming 20 years, the rates of mortality due to hepatitis will continuously increase. The difference between acute infection and chronic infection of HCV is that there is the presence of jaundice as its symptom, there is a history of an increase in the levels of ALT, and the duration of an increase in ALT. RNA might be spotted after 2 weeks of exposure to HCV in acute infections, while the antibodies against HCV can be detected after 2–3 months of virus exposure (50). The objective of this review is to assess the role of camel milk on multiple disorders of the liver.

In another study, the impact of camel milk to treat hepatitis C patients was investigated. To determine the liver functions of the patients, a half-liter of fresh pasteurized camel milk was given to each patient on alternate days. The study concluded that camel milk has a positive effect on the patients as it improves their total protein, albumin, and lymphocyte levels (63). In another study, camel milk's effectiveness in the treatment of patients with hepatitis C was determined. The serum of patients was collected before and after drinking camel milk and to evaluate the serum, three biomarkers ALT, AST, and anti HCV antibodies profile were observed. The results showed that the majority of patients have a positive effect on camel milk as the levels of AST and ALT were reduced after 4 months of drinking camel milk (58).

In another study, proteins in camel milk, i.e., camel polyclonal IgGs, and α-lactalbumin were separated and the antiviral activity against HCV was observed with the use of PBMCs and Huh 7.5 cell lines. The incubation of HCV with the purified proteins was done directly or the incubation of proteins with the cells was done before the exposure to HCV. The entry of HCV was inhibited by the interaction of HCV with camel IgGs and lactoferrin. The camel milk lactose Ferrin can inhibit the replication of HCV in cells at a specified amount. The consequences of this study conclude that the infectivity of HCV was repressed by lactoferrin in camel milk (64). In another study, lactoferrin was considered the primary pharmaceutical drug against HCV infection. The virus entry was inhibited with the direct interaction of camel lactoferrin and HCV after 7 days of incubation. Thus, the results conclude that lactoferrin has antiviral activity and an inhibitory role in the pathway of HCV infection and has more effectiveness than human lactoferrin (23).

### Hepatocellular carcinoma

The prime cancer of the liver is Hepatocellular carcinoma (HCC), worldwide, it is considered the fifth most common cancer and the third leading cause of mortality due to cancer. Its occurrence is common in many areas of the world as Asia, sub-Saharan Africa, as well as parts of Europe and the North American continent. In Egypt, the incidence rate of HCC in patients with liver diseases has increased over the past 10 years. In the occurrence of HCC, many risk factors such as inflammation have been implicated (65).

The beneficial potential of camel milk can be enhanced with the addition of cisplatin. The attribute of this positive therapeutic effect was due to the increase in the activity of superoxide dismutase and decrease in lipid peroxidation as well as a drop in the mean area of changed hepatocellular foci and the mean area of P-GST positive foci. The antioxidant effect of camel milk may have a role in the reduction of hepatocarcinogenesis when given cisplatin (56). In another study, El-Miniawy determined the impact of camel milk on the rats which were induced with hepatocarcinogenesis. The 28 rats were grouped into four groups having seven rats each. Diethyl-nitrosamine (DENA) injection was given to rats in the 6th week of camel milk treatment and after 34 weeks of injection, three rats were sacrificed from each group. The remaining rats were sacrificed on the 9th week of camel milk treatment after week 38. The levels of AST, ALT, albumin, and total protein in serum were examined spectrophotometrically. The levels of AST, ALT, albumin, total protein, and alpha-fetoprotein (AFP) in the serum of rats who were sacrificed were evaluated. Then the liver was examined histo-pathologically and alpha-fetoprotein was stained immunohistochemically and glutathione s transferase of the placental liver was carried out. Camel milk has a role in the liver protection against toxicity which was induced by DENA, as well as the progression to hepatocellular carcinoma was prevented, and the hepatocellular carcinoma growth was stopped (65).

**Figure 2:**
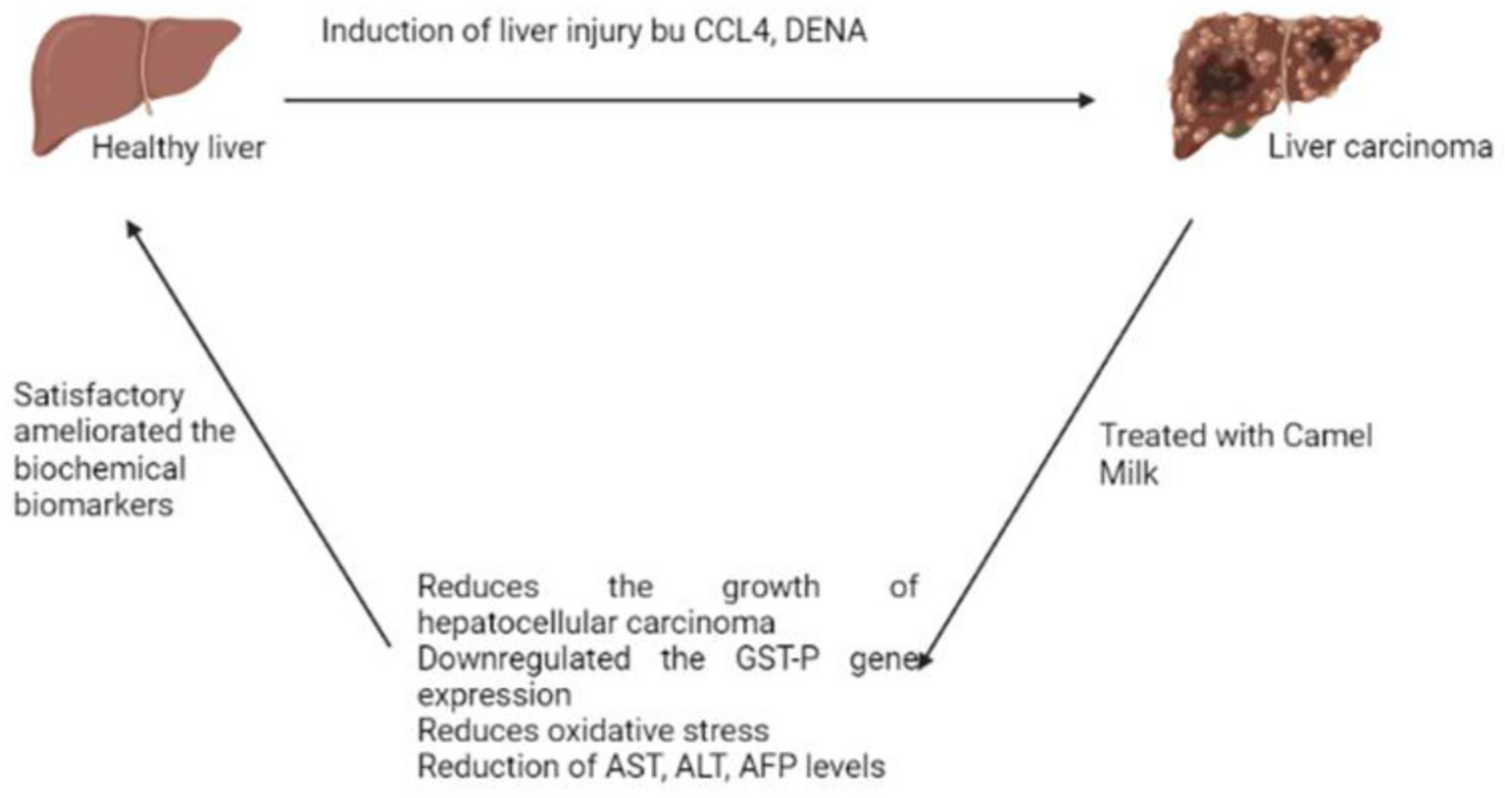
Effect of camel milk on liver carcinoma.

Moreover, the beneficial antitumor effects of antibodies in camel (*C. dromedarius*) milk (IgG) on DENA-induced carcinoma in liver cells in male Wistar rats were determined. Hepatocarcinoma was induced in rats using DENA (50 mg/kg, twice/week) for 2 weeks followed by CCl4 (1 ml/kg, trice/week) for 6 weeks. On week 17th, HCC-bearing rats were orally administrated camel milk IgG (100 mg/kg, orally) daily for 4 weeks. Liver enzyme activities and levels of alpha-fetoprotein (AFP) were measured in serum. Lipid peroxidation, and nitric oxide, reduced the levels of glutathione (GSH) were decreased, and the activity of superoxide dismutase (SOD) was determined in liver homogenate. Histological analysis using hematoxylin and eosin stain was examined in liver tissues. Hepatic mRNA gene expression of placental glutathione-s-transferase (GST-P) was determined by qRT-PCR. Treatment of HCC-bearing rats with IgG of camel milk significantly reduced liver injury biomarkers and attenuated oxidative stress as well as enhanced antioxidant status. Moreover, IgG camel milk significantly alleviated hepatocellular morphology alterations and down-regulated GST-P gene expression levels in the liver. The study concluded that there is an improvement in the dysfunction of the liver induced by DENA as well as in the oxidative stress in the rats having hepatocarcinoma was all attributed to the immunoglobulins purified from camel milk (46). Figure 2 explains the effect of camel milk on liver carcinoma.

### Alcoholic/non-alcoholic liver disease

Alcoholic liver disease (ALD) is a major cause of the increased number of disease cases and death rate worldwide. In Europe and the United States, ALD is considered one of the most common liver diseases, and ~300 million people are affected by hepatitis B virus, NAFLD, and ALD in China. ALD is characterized by a wide range of morphological features such as fatty liver—also known as steatosis—hepatitis, and alcoholic cirrhosis (39).

It is considered one of the major problems of liver diseases, and it is characterized by an imbalance of the intestinal microbes and hepatic inflammation. In the study, the rats having acute ALD were taken, and then the hepatoprotective effects of camel milk were investigated. Camel milk was given to different groups to determine the effect on the biochemical indicators. The hepatic inflammation in the ethanol plus camel milk group showed a reduction when the serum biochemical indexes and histology were analyzed. Camel milk suppresses the expression of genes related to inflammation such as (ILB and CXCL1) in the IL-17 and tumor necrosis factor (TNF-α) pathways. The results infer that hepatic inflammation and disorder in the microbial intestine, which were caused by acute alcohol injury were modulated with camel milk, which indicates the defending role of camel milk against liver injury induced by alcohol (39).

Moreover, the progression of NAFLD which was induced by a diet rich in fat and high fructose water was given to rats, and the preventive effects of FCM were investigated. Seven different groups were made and 42 male rats were divided into these groups randomly. A high-fat diet and high fructose in water (HFDHFr) were used to induce fatty liver disease. Camel milk was given as FCM or camel milk containing probiotic bacteria or containing probiotic without prebiotic, camel milk with 1% ginger extract, or microencapsulated probiotic with 10% beetroot extract. The serum of rats was analyzed to infer the activity of liver enzymes, insulin resistance, lipid profile, inflammation markers, and the stress due to oxidation. The study concluded that the concentration of serum glucose and the activity of liver enzymes were reduced with the intake of FCM-containing probiotics. Moreover, the groups that were given FCM containing probiotics and beetroot extract and camel milk with ginger extract have a positive impact on the liver (42).

**Figure 3:**
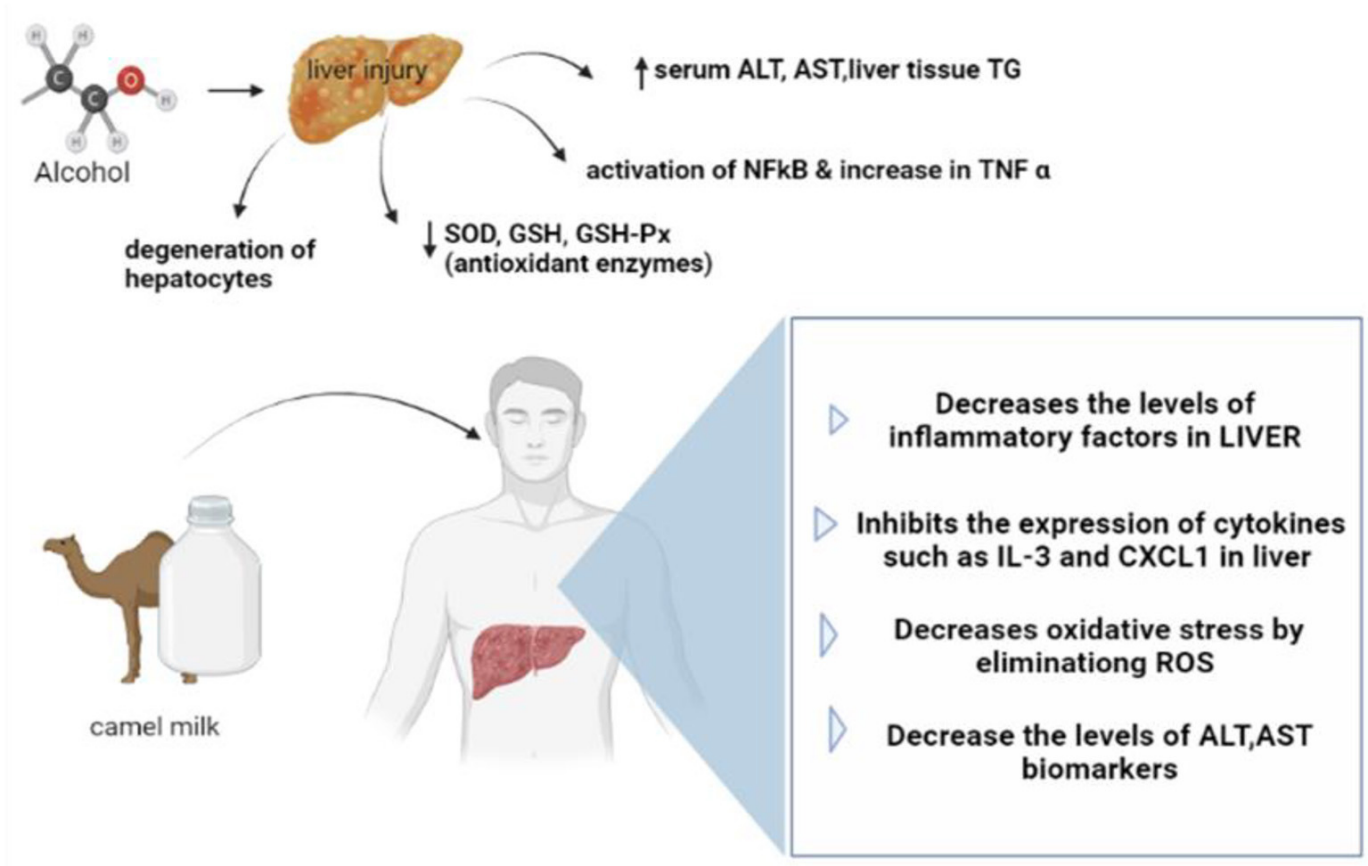
Effect of camel milk on alcoholic liver disease.

Another study was conducted on 30 male and female rats to evaluate the reactivation of liver functions due to the camel milk in the rats damaged by the Sudanese liquor (Aragi). The 24 rats were divided equally into two groups, control and tested. The levels of Glutamate Oxaloacetate Transaminase (GOT), Glutamate Pyruvate Transaminase (GPT), and Alkaline Phosphatase (ALP) were measured for both groups. Liver samples were investigated and it infer that camel milk reduced the level of these enzymes to their normal. The reductions in the levels of these enzymes from day (30) to day (60) were 73.2, 53.9, and 65.4%, respectively. It was concluded that camel milk can be used as a herbal treatment for alcoholic diseases as well as in the treatment of other liver diseases which may have an impact on the liver enzymes and other tissues (66). Figure 3 presents the effect of camel milk on alcoholic liver disease.

### Hepato-toxicity

To assess how camel milk impacts newborn rats who are given ethanol to induce hepatotoxicity, a study was conducted. The rats were divided into five groups having six rats in each group. There is a significant rise in the levels of MDA and the activity of SOD, CAT, and GSH-PX enzymes were reduced in the liver tissues of newborn rats as well as there are histological changes in the tissues of the liver when they are treated with ethanol during pregnancy. Moreover, when this group was related to the control group the activity of serum enzymes was increased. The study concludes that there are protective and preventative effects of camel milk on newborn rats who are induced with hepatotoxicity (45). In another study, the toxic properties of CCL_4_ on the tissue of the liver were examined with exposure to camel milk and camel urine. Liver enzymes including ALT, AST, and ALP were monitored as there is an increase in their values when a single dose of CCL_4_ was given to them. The four groups were made and rats were divided equally, and then each group was given a different diet the first group was the control group to which standard diet and tap water were provided. The second group was named as a positive control group and given 1 ml/kg of BW of CCL_4_ with the same ratio of 1:1 of olive oil for 4 weeks. The third group was given camel milk (100 mL/day) with a normal diet and CCL4 (1 ml/kg of BW) was injected and the hepatoprotective effect was tested. The fourth group was fed with camel urine (100 mL/day) with a normal diet and CCL_4_ (1 ml/kg of BW) was injected. The blood samples of rats were collected and analyzed. The serum was separated by centrifugation and the serum activities of ALP, AST, and ALT were monitored to detect the hepatotoxicity caused by CCL_4_. The study concluded that the group which was given camel milk had a positive effect in improving the hepatotoxicity caused by CCL_4_. The study also concludes that camel urine has a protective effect against toxicity (34).

Moreover, camel milk contains lactoferrin which has antiviral, as well as anti-inflammatory properties. It can be utilized to treat hepatic fibrosis in Sprague Dawley rats caused by CCL4. Five groups were made by dividing 75 male Sprague–Dawley rats randomly. Carbon tetrachloride (CCL_4_) was provided to each group at 200 uL/100 g BW as a single dose with a mixture of 40% CCL_4_. The standard diet was provided to the control group while the remaining four groups were provided orally with lactoferrin along with the standard diet. It was a two phase study in which in the initial phase, the camel milk lactoferrin was isolated and purified. In the next phase, lactoferrin's efficacy against carbon tetrachloride-induced liver toxicity in Sprague Dawley rats was investigated. The study concludes that lactoferrin in camel milk may cause improvements in the liver toxicity induced by CCL_4_ in rats illuminates that there are improvements in the serum levels of ALP, AST, AST, bilirubin, serum urea, and serum creatinine in 4 weeks of treatments with camel milk lactoferrin (40).

Similarly, Camel milk and *Nigella sativa* oil (NSO) have anti-hepatotoxic potential against hepatic and nephrotoxicity induced by TAA in rats was investigated. The study was conducted on rats and six groups were made of 30 female rats that were divided equally into these groups having five rats in each group. To induce hepatorenal toxicity, on the first day, all animals from Groups II to VI received a single injection of TAA (100 mg/kg BW) in the form of a 2% w/v solution. The histopathological investigations were done on the preserved tissues of the liver and kidney. Commercial diagnostic kits were used to determine biochemical parameters such as serum levels of AST, ALT, GGT, ALP, uric acid, urea, creatinine, salt, and potassium. To check the changes, histopathological assessments of liver and kidney tissues were assessed according to the standard method. In comparison to the normal control rats, a single injection of TAA (100 mg/kg) was administered by injection that will increase the blood levels of ALT, AST, ALP, and GGT, confirming the initiation of hepatotoxicity. When the experimental animals are treated with camel milk, NSO alone or its combination may increase the levels of AST, ALT, and ALP and improve the liver toxicity induced by TAA. The results concluded that camel milk, NSO, and camel milk in combination with NSO are effective in correcting toxicity of liver and kidney in rats (41).

Likewise, in gamma-irradiated Albino rats, the preventive properties of camel milk were examined. The 96 Sprague Dawly healthy adult male albino rats were classified into 16 groups and then labeling was done. The parameters of hepatological and nephrological were biochemically measured in the blood sample that was collected. The examinations of the liver and kidney were done. There will be changes in the functions of the liver and kidney along with changes in protein profiles caused by IRR. There will be dose-dependent reduction in the levels of serum protein such as total soluble proteins, and the levels of albumin and globulin. There will be an increase in the serum levels of bilirubin, urea, uric acid, ALT, AST, ALP, and creatinine levels significantly. Camel milk therapies in IRR rats restored their damaged status and there will be a reduction in the changes in liver and kidney functions, as well as hematological abnormalities related to IRR's adverse effects (3). Similarly, there is a restoration effect of camel milk on the liver toxicity induced by methoxychlor (MXC). Methoxychlor is an environmental contaminant, that is commonly used in many countries as a pesticide, and here it has been used for the induction of liver toxicity in rats. There is a significant increase in the levels of serum transaminases (AST and ALT) and alkaline phosphatase when MXC is given to rats, while there is a significant decrease in the levels of total protein and albumin. Lipid peroxidation is inhibited with MXC and it causes the elevation of glutathione levels in the liver homogenate. In the liver, pathological damages such as degradation of hepatic cells and coagulative necrosis were discovered. The daily dose of 100 ml/day in each cage of the camel milk-treated group was given and it was the only source of drinking for them for 6–12 months. On the other hand, an oral dose of MXC 200 l/kg of BW two times a week was given to the MXC-treated group, for the same period. The study concluded that there is a reduction in the deterioration of liver cells and normal structure of other cells, as well as the liver histopathological analysis, which was inconsistent with the biochemical findings. These effects proved the hepato-protective role of camel milk (35).

### Cirrhosis

A study was conducted on male rats to investigate the impact of camel milk in combination with bee honey against hepatotoxic compounds. Two main groups were made and 36 rats were divided into these groups. The first group was the control group having *n* = 9 non-cirrhotic rats. The rats *n* = 27 in the other group were given carbon tetrachloride injection for the induction of cirrhosis. It was determined that the activities of enzymes of the liver, blood glucose level, non-esterified fatty acids (NEFA) in the serum, and glycogen amount in the liver increased with CCL4. Similarly, in the liver tissues, the activity of the phosphorylase enzyme is reduced, and elevated carbohydrate intolerance as well as the resistance index of insulin. Furthermore, CCL_4_ has an elevated impact on the expression of TNF-α and TGF-β which are cytokine genes, and it induces the elevation of oxidative stress. However, camel milk can improve the toxic effects either alone or in combination with bee honey. This protection is based on the antioxidant capabilities of these preventive compounds and their impact on downregulating specific pro-cirrhotic cytokine gene transcripts (52).

In a study, Egyptian patients who are infected with HCV were enrolled having infection in the parenchyma which is mild to moderate and followed by mild cirrhosis after taking their history and clinical examination. To check the effect of camel milk, it is given to the patients, and then their biomarkers were evaluated. The marked inhibition of serum levels of the inflammatory biomarkers showed the improving effect of camel milk. The study concluded that camel milk has a regulatory function on the multiple parameters of mediators of inflammation, modulators of the immune system, antiapoptotic, and antioxidants, which infers the potential therapeutic advantage of camel milk against HCV (67).

## Conclusion

The present study highlighted the protective and therapeutic role of camel milk on multiple liver disorders. People who consume camel milk have a significant improvement in the lab values of serum gamma-glutamyl transferase, aspartate aminotransferase, and alanine aminotransferase. The proteins in camel milk are heat-stable even at high temperatures and remain functional. It is therefore immensely important to understand the special attributes of camel milk and make it possible to fully utilize its potential. The prospect includes the utilization of camel milk as a nutraceutical agent and makes it possible to easily avail this in markets because it is a good choice for people who are lactose intolerant. So, the consumption of camel milk alone or in combination with any nutraceutical should be encouraged.

## Author contributions

Conceptualization: KS, I-U-H, RR, and RA. Writing—original draft preparation: KS, RR, and SS. Writing—review and editing: AN, MM, NW, CS, CM, and RA. Supervision: RA. All authors have read and agreed to the published version of the manuscript.

## Conflict of interest

The authors declare that the research was conducted in the absence of any commercial or financial relationships that could be construed as a potential conflict of interest.

## Publisher's note

All claims expressed in this article are solely those of the authors and do not necessarily represent those of their affiliated organizations, or those of the publisher, the editors and the reviewers. Any product that may be evaluated in this article, or claim that may be made by its manufacturer, is not guaranteed or endorsed by the publisher.
